# Histological study on maturation, fertilization and the state of gonadal region following spawning in the model sea anemone, *Nematostella vectensis*

**DOI:** 10.1371/journal.pone.0182677

**Published:** 2017-08-10

**Authors:** Elizabeth Moiseeva, Claudette Rabinowitz, Guy Paz, Baruch Rinkevich

**Affiliations:** Israel Oceanography and Limnological Research, National Institute of Oceanography, Tel-Shikmona, Haifa, Israel; University of California Irvine, UNITED STATES

## Abstract

The starlet sea-anemone *Nematostella vectensis* has emerged as a model organism in developmental biology. Still, our understanding of various biological features, including reproductive biology of this model species are in its infancy. Consequently, through histological sections, we study here key stages of the oogenesis (oocyte maturation/fertilization), as the state of the gonad region immediately after natural spawning. Germ cells develop in a secluded mesenterial gastrodermal zone, where the developing oocytes are surrounded by mucoid glandular cells and trophocytes (accessory cells). During vitellogenesis, the germinal vesicle in oocytes migrates towards the animal pole and the large polarized oocytes begin to mature, characterized by karyosphere formation. Then, the karyosphere breaks down, the chromosomes form the metaphase plate I and the eggs are extruded from the animal enclosed in a sticky, jelly-like mucoid mass, along with numerous nematosomes. Fertilization occurs externally at metaphase II via swimming sperm extruded by males during natural spawning. The polar bodies are ejected from the eggs and are situated within a narrow space between the egg’s vitelline membrane and the adjacent edge of the jelly coat. The cortical reaction occurs only at the polar bodies’ ejection site. Several spermatozoa can penetrate the same egg. Fertilization is accompanied by a strong ooplasmatic segregation. Immediately after spawning, the gonad region holds many previtellogenic and vitellogenic oocytes, though no oocytes with karyosphere. Above are the first histological descriptions for egg maturation, meiotic chromosome’s status at fertilization, fertilization and the gonadal region’s state following spawning, also documenting for the first time the ejection of the polar body.

## Introduction

*Nematostella vectensis* Stephenson, 1935 (Actinaria, Edwardsiidae), is a small widespread burrowing sea anemone living in estuaries along the Atlantic and Pacific coasts of North America and the southeastern coast of England [1–3]). With a sequenced genome, *N*. *vectensis* has emerged as a model organism in developmental biology and metazoan evolution [4–9], as well as in ecological and toxicological studies [10–12].

Studies on *N*. *vectensis* in the last decade have focused on the origin of bilateral symmetry [13,14], the evolution of the mesoderm [15–17], the signaling pathways and the mechanisms of regeneration [18–27] the development of the nervous system [25], pattern formation [27] and more. However, our understanding of various biological features, including reproductive biology characteristics of this powerful model species are in its infancy.

*N*. *vectensis*, a gonochoristic species, spawns routinely under laboratory conditions [28]. Under different combinations of photoperiods, temperatures and diets it may spawn continuously throughout the year, forming mature eggs every 7–8 days [29]. Oocytes, which develop and mature in the mesenteries, are extruded through the mouth while immersed in a jelly coat as a part of the egg masses [5, 28, 29].

Primordial *N*. *vectensis* gametes were identified during embryogenesis and early development by exploring the expression of the *Vasa* and *Nanos* gene [30]. Histological data regarding developing oocytes and mature eggs [31, 32], including electron-microscopy descriptions [5, 33], have provided only slim information about oocyte growth and vitellogenesis. Attempts to determine the meiotic status of the chromosomes during egg ovulation and fertilization were unsuccessful [32, 34], and we are not familiar with studies that use histological descriptions of maturation and fertilization of the germ cells in *N*. *vectensis*. Lee et al. [35] documented two polar bodies during fertilization, but these observations were not illustrated in details. Thus, until now, the meiotic status of matured *N*. *vectensis* eggs during fertilization remains unclear, since no polar body ejection has been documented and the fertilization process was not shown histologically. Also, there is no detailed documentation of the morphological state of the gonadal region immediately following spawning.

This study thus aims to histologically investigate oocyte development during maturation and fertilization, as well as the structure and the composition of female sex cells in the gametogenic area of the mesentery immediately after spawning.

## Materials and methods

Adult *Nematostella vectensis* individuals were cultivated according [36], with minor modifications according to [27, 28]. *N*. *vectensis* culture conditions are detailed in [37]. Males and females were reared in the same dishes (100x20 mm, cell culture dishes, cat. 664.160, Cellstar, Greiner bio-one, Germany) and gametes were taken as a result of natural spawning. Gravid females were fixed before spawning, at the onset and immediately after spawning. The animals were narcotized by adding 7.5% MgCl_2_ to the culture dish and then fixed in Bouin’s solution for 60–120 min or in 4% buffered formalin for 24 hours, at room temperature. Eggs were fixed at the moment of their extrusion from the female body as well as 3–5 minutes later in Bouin’s solution for 15–30 min. After fixation, the samples were dehydrated in a graded ethanol series (70–100%), in a mixture of ethanol:butanol (I:I) and in 100% butanol and embedded in Paraplast (cat. P3683 Sigma Aldrich, St. Louis, MI, USA). Serial sections (4–5μm thick) were prepared using a rotary microtome (cat.2045, Jung Multicut, Leica). After staining, the sections were embedded in Entellan (Merck, Germany). Slides were observed under an Olympus BX50 upright microscope, equipped with a Color View camera (Soft Imaging System, Munster, Germany). A total of 7 adult females and 47 eggs from 3 egg masses were examined.

We used Mayer’s alum hematoxylin and eosin (AHE) staining protocol to perform a standard histological examination, a periodic acid-Schiff’s reagent (PAS-method) counterstained with alum hematoxylin (PAS-AH) for polysaccharides and an amylase treatment on parallel sections as a control for glycogen; paraldehyde fuchsin—alcian blue (pH 2.8–3.0) (PAF-AB)–was used to identify mucins (‘Steedman’s method’) [38], in combination with eosin or azocarmine G. For mucopolysaccharides we used alcian blue with the PAS-method and alum hematoxylin (AB-PAS-AH) [39]; for nuclear staining either DAPI or ethidium bromide (EtBr) were used.

Immunolabeling for phospho-histone H3 (ser 10-R; specificity checked in ref. 23) was performed using commercial rabbit polyclonal antibodies (Santa Cruz Biotechnology, USA). Blocking for nonspecific binding sites was performed with 1% bovine serum albumin dissolved in 50 mM TBS for 4 h incubations at room temperature. The blocking solution was replaced with commercial rabbit polyclonal-primary-antibodies (Santa Cruz biotechnology, USA) diluted in blocking buffer (1:400) for an overnight incubation, followed by a 2 h incubation with a goat anti-rabbit secondary antibody (1:400), conjugated to the fluorescence Cy3^™^- AffiniPure, Goat anti-Rabbit IgG Amax-550 and Emax-570 (Cat No. 111-165-003; Jackson ImmunoResearch Laboratories, Pennsylvania, USA).

All slides were mounted in a Fluoromount medium (cat F4680; Sigma, Germany) and photographed using the Olympus Bx50 upright fluorescent microscope.

## Results

### General morphology

**Fig 1:**
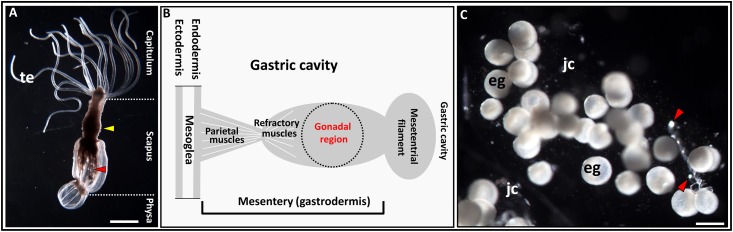
General view of the adult *Nematostella vectensis*. **A.** Three main body parts are shown. Yellow arrowhead indicates the location of the gonadal region within the body; red arrowhead indicates the mesentery within the scapus. **B**. A schematic drawing of the gonadal region localization within the mesentery in transverse section. **C**. The egg masses with eggs and the nematosomes (arrowheads). eg, egg; jc, jelly coat; te, tentacles. Scale bars: A = 2.0 mm, C = 200 μm.

**Fig 2:**
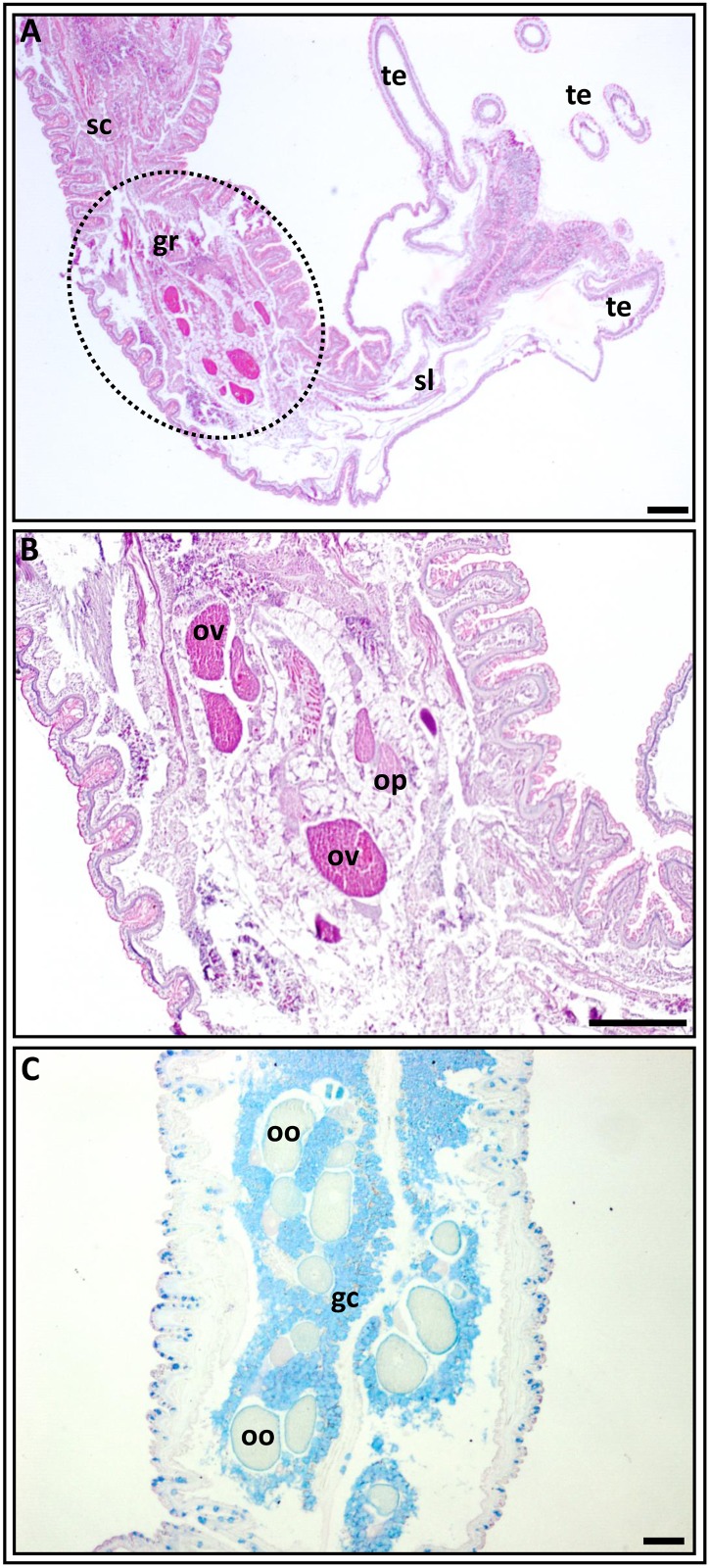
Longitudinal sections through a gravid *Nematostella*’s mesentery. **A**. The upper part with tentacles (te), the scapulus (sl) and part of the scapus (sc) with the gonadal region (gr) (outlined) are seen. **B.** A higher magnification of the outlined zone in “A” reveals germ-cells groups with vitellogenic (ov) and previtellogenic (op) oocytes, and glandular cells surrounding them. Vitellogenic oocytes are strongly stained with azocarmine. PAF-Az stain. **C.** The germ-cells groups in the gonadal region, which contain selectively colored AB-positive glandular cells (gc) surrounding colorless oocytes (oo), can be seen. PAF-AB stain. gc, glandular cells; oo, colorless; oocyte; op, previtellogenic oocyte; ov, vitellogenic oocyte; sc, scapus; sl, scapulus; te, tentacles. Scale bars = 100 μm.

**Fig 3:**
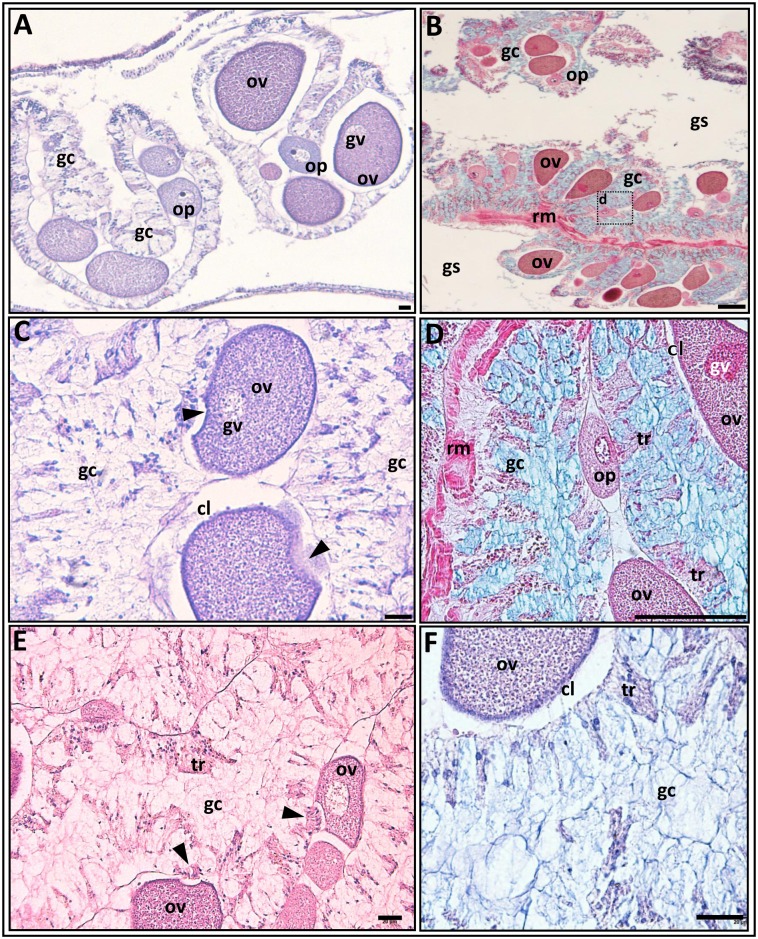
The gonadal region immediately after the extrusion of an egg mass. **A.** A general view of the gonadal region in a cross section. Germ-cells groups are well visible. AHE stain; **B**. The longitudinal section of the gonadal region part. The gastric cavity (gs) is empty and clearly visible. Groups of germ cells at different stages of oogenesis and the glandular cells (gc) that surround them are seen within the gonadal region. PAF-AB-Az stain. **C**. Vitellogenic oocytes (ov) with an invagination of the oolemma (arrowhead). AHE stain. **D.** The part of the gonadal region seen in **‘B’** at a higher magnification. Note the bands of trophocytes (tr) between the glandular cells. PAF-AB-Az stain. **E,F.** Glandular cells show a reduced intensity of staining with reagent Schiff (PAS-AH stain in **E**) and alcian blue (AB-AHE stain in **F**) as compared to pre-spawning stage (Fig 4B and 4C; in both dyes, the fixation and staining periods were identical). In **‘E’** the vitellogenic oocytes with an invagination of the oolemma and trophonema (arrowheads) are seen. cl, cortical layer; gc, glandular cells; gs, gastric cavity; gv, germinal vesicle; op, previtellogenic oocyte; ov, vitellogenic oocyte; rm = retractor muscle; tr, trophocytes. Arrowheads in **C** show invagination of the oolemma; arrowheads in **E** show trophonema. Scale bars = 20 μm.

**Fig 4:**
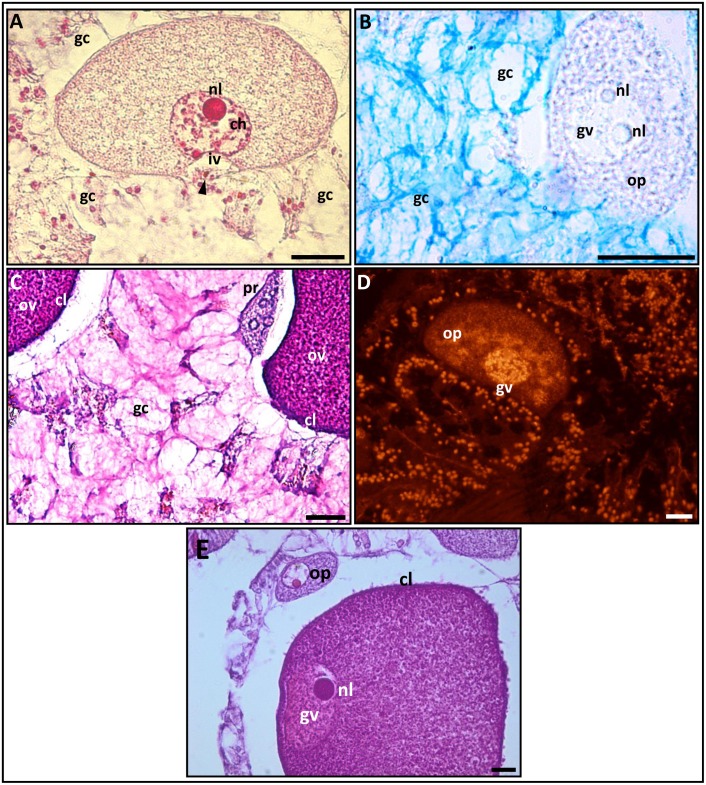
Oocytes of the different stages of oogenesis and the glandular cells surrounding the oocytes within the germ-cells groups. **A**. The polarized previtellogenic oocyte, characterized by lamp-brush chromosomes (ch) inside the germinal vesicle, the oolemma’s invagination (iv) and the trophonema (arrowhead), which is well developed in the site of invagination. PAF-Az stain. **B,C**. Alcian blue-positive (**B**) and PAS-positive (**C**) glandular vacuolated cells (gc). The previtellogenic oocyte (op) (colorness in ‘B’) with nucleoli (nl) inside germinal vesicle (gv) and the vitellogenic oocytes (ov) are filled of the yolk granules (in ‘**C**’) can be seen. PAF-AB and PAS-AH stain, respectively. **D**. Histone H3 phosphorylation in prophase I meiosis chromatosomes in the germinal vesicle of a previtellogenic oocyte. **E.** The oocyte of the end of vitellogenesis. The germinal vesicle is located at the animal pole under the cortical layer (cl) of the ooplasm, and the ooplasm is filled with yolk granules and globules. The external edge of the cortical layer is visible”rough”. ch, chromosomes; cl, cortical layer; gc, glandular cells; gv, germinal vesicle; iv, invagination of the oolemma; nl, nucleoli; op, previtellogenic oocyte; ov, vitellogenic oocyte. Arrowhead shows trophonema (in **A**) AHE stain. Scale bars: A-D = 20 μm, E = 10 μm.

**Fig 5:**
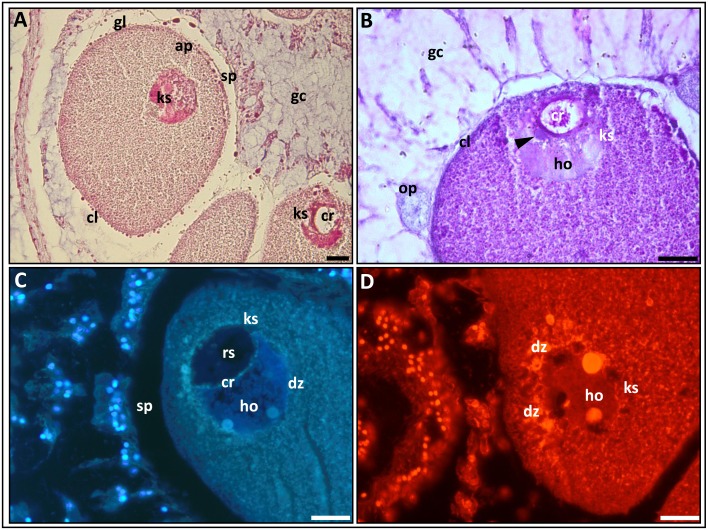
The karyosphere (ks) in maturating oocytes. **A**. A general view of an oocyte with karyosphere. The yolk globules (gl) along the edge of the cortical layer (cl) and the space (sp) between the oolemma and the gastrodermal cells are seen. PAF-Az stain. **B.** Three karyosphere zones are visible: a relatively homogenous substance (ho), a denser, lumpy substance (arrowhead) and a ring-shaped zone. The chromosomes (cr) are inside the ring-shaped zone. AB-PAS stain. **C.** Condensed chromosomes (cr) are in the dense zone of the karyosphere. Note the space (sp) between the oolemma and the gastrodermal cells. DAPI stain. **D**. Chromosomes are in dense zone of the caryosphere during condensation. EtBr stain. cl, cortical layer; cr, chromosomes; dz, dense zone; gc, glandular cells; gl, yolk globules; ho, gomogenous zone; ks, karyosphere; op, previtellogenic oocyte; rz, ring-shaped zone; sp, space between the oolemma and gastrodermal cells. Arrowhead shows the dense zone of the karyosphere (in **B**). Scale bars = 20 μm.

The morphological features of *N*. *vectensis* individuals have been previously described [1,2,16,40]. General view of the adult animal is shown in Fig 1A. It is evident that this sea anemone has no true ovaries [5], whereas a “gonad” in *N*. *vectensis* is the determined gametogenic area of the mesentery, which is located between the retractor muscles and the mesenterial filaments (Fig 1B). This zone can be further differentiated from other mesenterial areas by its cytomorphological characteristics: it hosts the germ cells exclusively (Fig 2A), so in this article we use the term “gonadal region”. The general morphology of the gonadal regions of the pre-spawning and post-spawning anemones do not differ, with the exception of the sex cells composition. In order not repeat description, we considered it expedient to simultaneously describe the general morphology for both pre-spawning and post-spawning individuals. The gonadal region consists of several local germ-cells groups (>10 groups/middle cross section), (Figs 2B, 2C, 3A and 3B). Inside one group oocytes are developed asynchronously, so all stages of oogenesis are represented from small previtellogenic oocytes to large vitellogenic and maturing ones (Figs 2B, 3A, 3B, 4 and 5). In addition to oocytes of different stages of development, the gonadal region possesses the vacuolated gastrodermal cells (glandular elements), forming a loose network around germ cells (Figs 2C and 3B–3E), as well as the trophocytes (accessory cells) (Fig 3D–3F). Glandular cells are stained by alcian blue (Figs 2C, 3D, 3F and 4B and positively stained by Schiff-reagent (Figs 3E and 4C), thus revealing the existence of various polysaccharides, mucopolysaccharides and mucoproteins. When using the AB-PAS stain, the vacuolar membrane and the filaments within the vacuoles are stained light green and pink, respectively (Fig 4B and 4C). AHE reveals that the vacuole’s content does not stain with hematoxylin, and so it appears to be a light pinkish hue (Fig 3C). Furthermore, the glandular cells’ PAS-positive material retains its color after the 1% amylase treatment (30 min, room temperature), indicating it is not a glycogen. Trophocytes, which are groups of dense, small cells, are situated between the glandular cells. During growth and development of oocytes, they are formed in the trophonema which is a unique association of germ and somatic cells [5]. The trophonema is clearly visible in large polarized previtellogenic and vitellogenic oocytes and is distinct in oocytes remaining in the gonad region after spawning. In the trophonema location the invagination of outer ooplasmatic layer can be seen (Figs 3C, 3E and 4A).

### Maturation

At the end vitellogenesis the large polarized oocytes reach a diameter 130–160 μm, germinal vesicles are 50–70 μm. Germinal vesicles are located in the region of the animal pole. The karyoplasm can be strongly stained by eosin; yolk inclusions are evenly distributed throughout the ooplasm (Fig 4E). They are intensively stained by eosin and show a strong PAS-reaction (Fig 4C and 4E) that remains following the treatment with amylase. The cortical layer of ooplasm is well defined (Figs 3C, 3E, 3F and 4C) and its external edge is visible “rough” (Fig 4E)

The oocyte’s transition into maturation process is characterized by the formation of the karyosphere at the animal pole of the cell (Fig 5). The karyosphere is a modified germinal vesicle. During the formation of the karyosphere a gradual condensation of chromosomes occurs. The germinal vesicle loses its normal morphology and is transformed into an amorphous structure, the location of which is distinctly separate from other zones of oocyte (Fig 5A). The karyosphere’s form is variable, and most often it is composed of three parts: a relatively homogeneous light substance, a denser, lumpy substance and a ring zone (Fig 5B and 5C). The chromosomes, which can be located at any part of the karyosphere, are very condensed. They form crystalline lumps and display the same weak PAS-reaction as the yolk granules. DAPI and EtBr staining are usually observed in the dense crystalloid formation (Fig 5C and 5D). Yolk inclusions are evenly located in the ooplasm, with no visible gradient in distribution between the animal and the vegetative poles (Fig 5A). The cortical layer stains strongly and its external layer appears rough (Fig 5A), like in the large vitellogenic oocyte (Fig 4E). The cortex’s granules are marked by a darker color, which strongly differs from the color of the glandular cells that surround the germ cells (Fig 5A and 5B).

**Fig 6:**
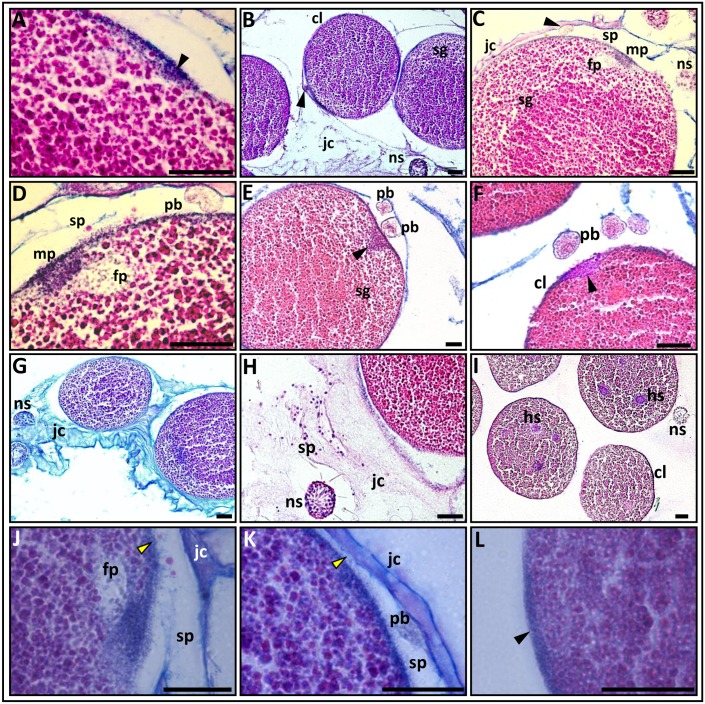
Mature eggs during fertilization. **A**. The metaphase II plate is located at the animal pole of the egg (arrowhead); AB-PAS-AH stain. **B.** The first polar body (arrowhead) that appeared in the space between the oolemma and edge of the jelly coat (jc). The segregated ooplasm (sg) is seen. AB-AHE stain. **C.** A general view of the animal pole during fertilization. The first polar body (arrowhead), the male pronucleus (mp), the female pronucleus (fp), the space (sp) between the oolemma and the edge of the jelly coat and the segregated ooplasm are seen. PAS-AB-AH stain. **D**. A higher magnification of the animal pole shown in **‘C’**. The polar body (pb) is clearly seen in a state of division. **E,F.** The formation of two (**E**) and three polar bodies **(F**). The male pronucleus (arrowhead) is located in the peripheral zone of the egg (the coarse-grained yolk area) and the segregated ooplasm (in ‘**E**’) are clearly visible. AB-PAS-AH stain. **G**. Eggs located within the jelly coat. The ooplasmatic segregation is clearly visible. Inside the jelly coat are nematosomes (ns). AB-AHE stain. **H.** Numerous spermatozoa (sz) and one nematosome are seen inside the jelly coat. AHE stain. **I.** Some sperm heads (hs) are seen inside the eggs. They are visible as dark spots. AHE stain. **J.K.** The edge of the cortical layer (arrowheads) at the animal pole is characterized by a rough view. Fine granules are seen in the space between the oolemma and the internal edge of the jelly coat. In **‘J’** the female pronucleus is clearly seen, in **‘K**’ the polar body is depicted. **L.** The edge of the cortical layer at the vegetative pole of the same egg is smooth (arrowhead). AB-AHE stain. cl, cortical layer; fp, female pronucleus; hs, sperm heads; jc, jelly coat; mp, male pronucleus; ns, nematosome; pb, polar body; sg, segregated ooplasm; sp, space between the oolemma and the edge of the jelly coat; sz, spermatozoa. Arrowhead in **A** shows metaphase II plate; arrowheads in **B** and **C** show the polar bodies; arrowheads in **E** and **F** show male pronuclei; yellow arrowheads in **J** and **K** illustrate a rough view of the cortical layer edge at animal pole and arrowhead in **L** shows a smooth view of the cortical layer at the vegetative pole in the same egg. Scale bars = 20 μm.

By the end of the maturation process the chromosomes progress from stage of the prophase meiosis I to the metaphase stage. The metaphase plate lies just below the surface of the oocyte, among the yolk globules, and is barely visible. Ovulation takes place in the gastrovascular cavity in concert with the formation of the protective jelly coat, and then the eggs together with the numerous nematosomes, containing nematocyst capsule, are extruded from maternal organism in the form egg mass (Fig 1B). The jelly coat is made from a variety of mucins all with tinctorial properties similar to the content of the gonadal region’s glandular vacuolated cells (Fig 6B, 6G and 6H).

### Fertilization

The diameters of the fertilized eggs range between 158 and 175μm. Fertilization occurs when the chromosomes are in the metaphase II stage (Fig 6A), concurrent to the emission of the first polar body, as revealed by the simultaneous presence of the polar body and the spermatozoa or male pronucleus (Fig 6C, 6D and 6H). The first polar body (about 25μ) is larger than the second body and contains a small amount of chromatin (Fig 6D–6F). The male pronucleus passes through the oolemma and moves in the direction of the female pronucleus. As the nuclear membrane of the fertilizing spermatozoa dissolves, the male pronucleus is revealed as an amorphous, borderless, finely-granular structure (Fig 6C–6F). The female pronucleus looks like a light bubble with condensed chromosomes inside (Fig 6C, 6D and 6L). We have documented cases where several spermatozoa penetrated the egg, they can be seen as dark spots (with dissolved head membranes) inside the ooplasm (Fig 6L). During fertilization the adjacent internal layer of the jelly coat is separated from the oolemma exactly at the animal pole’s location, allowing the polar body to be shed (Fig 6B). The perivitelline space appears only at the animal pole, suggesting that the cortical reaction takes place precisely in this zone. The outermost edge of the oolemma at the fertilized egg’s animal pole looks very uneven and rough (Fig 6L and 6K), while the edge of the same egg’s vegetative pole has a smooth outline (Fig 6L). The spermatozoa’s penetration into the egg is accompanied by ooplasmic segregation, during which fine and small yolk inclusions move and concentrate into the egg’s central zone while large granules and globules of yolk remain at its periphery. As a result, the central zones appear more densely stained than the peripheral zones (Fig 6C, 6F, 6E and 6G). The ooplasmic segregation is crucial for following the spatial organization of the embryo tissues [38].

### The morphology of the gonadal region immediately after spawning

After the egg mass has left the female body, left over germ-cells groups within the gonadal region become clearly visible (Fig 3A and 3D), containing in each group previtellogenic and vitellogenic oocytes of different sizes that are also surrounded by gastrodermal glandular cells (Fig 3B–3E). The larger polarized previtellogenic oocytes are marked by an invagination of the oolemma, which occurs at the same location as the trophonema (Figs 3C, 3E and 4A). The nearly colorless nucleoplasm contains rough chromosome threads (lampbrush chromosomes) and 1–2 large eccentric nucleoli (Fig 4A). In some oocytes H3 phosphorylation is well expressed suggesting that the chromosomes are in prophase I meiosis (Fig 4D). The vitellogenic oocytes of different sizes contain varied amounts of yolk inclusions, which stain strongly with eosin and give PAS-reaction that persists after amylase treatment (Fig 3C and 3E). In all cases observed the largest remaining vitellogenic oocytes in the gonadal zone do not yet contain the karyosphere, although the invagination of oolemma in the animal pole is noticeable, the cortical layer is well defined and the germinal vesicles are located near this invagination (Fig 3C and 3E). The abundance of glandular cells does not vary noticeably, but the staining of the cells is less intensive, as a result of a certain amount of secretory substances that have already been released together with the eggs (Fig 3E and 3F). The trophocytes appear as longitudinal strands located between groups of glandular cells (Fig 3D–3F)

## Discussion

In this study key stages of the oogenesis (maturation and fertilization) of *Nematostella vectensis* and the state of the gametogenic region at the mesentery immediately after spawning are studied using light microscopy with different staining approaches. These stages of oogenesis are described for the first time, as are the identification of the chromosomal status at the moment of fertilization and the documentation of the polar bodies. These findings supplement the information currently available regarding the reproductive biology of *N*. *vectensis*, a model species used in studies on metazoan development.

As mentioned above, *N*. *vectensis* has no true ovary and its sex cells develop in a gametogenic region of the mesentery [5], like other anthozoans [41, 42]. In sea anemones (Cnidaria, Anthozoa) “the term gonad is conditionally applied to the strips of tissue within the mesenteries where sexual products accumulate” [42, 43]. Describing of the local germ cells groups within gametogenic region of the mesentery, authors used different terms. So, Wilcox [34] called such structures in *N*. *vectensis* as “cysts” while Eckelbarger and Larson [44, 45] used the term “islands” in *Aurelia aurita*. We used the term “germ-cells groups”. In these groups oocytes develop asynchronously, as is typical for many species of sea anemones [43], including *N*. *vectensis* [5, 34]. The maturation of large, polarized, vitellogenic oocytes begins with the germinal vesicle transformation to the karyosphere. The karyosphere, a specific structure that consists of composite multiple complexes of nuclear and cytoplasmic proteins [46, 47], protects the oocyte’s genetic apparatus and decreases or completely stops its synthetic activity during the maturation process [48]. The formation of karyosphere during maturation is documented in different species of invertebrates and vertebrates [40–56]. In some animals, a decrease of the chromosomal transcriptional activity can be observed during the formation of the karyosphere and a complete cessation has been seen in the ring-shaped karyosphere. This was documented in insects [47, 48] as in the humans’ preovulatory oocytes [51]. The morphological structure of the *N*. *vectensis* karyosphere is very similar to the karyosphere found in the insect *Tenebrio molitor* [48].

Based on the morphological features of the karyosphere in *N*. *vectensis* it is further assumed that chromosomes’ synthetic activities die away near the end of the maturation process. This assumption is indirectly confirmed by the almost complete absence of visual contacts between the maturating oocytes and gastrodermal cell, where empty spaces are seen between them (Fig 5A and 5C compare with Fig 4A–4C).

The mature oligolecithal *Nematostella* egg contains a small amount of yolk that is evenly distributed throughout the ooplasm (isolecithal type, following [57]). In the present study, the dimension of eggs inside the egg masses varied between 158μ and 175μ., while Wilcox [34], Frizenwanker et al. [32] and Lee et al. [35] observed larger eggs (170μ–200μ; 210μ–270μ; 198μ–247μ, respectively). It is, however, well documented that cell sizes tend to decrease in the course of histological processing [38, 39]. Fertilization occurs at chromosomal status metaphase-II, outside the female body. The ejection of the first polar body, the fertilization and the segregation of the ooplasm occur almost simultaneously, as evidenced from the location of the male pronucleus (either inside the egg or on its surface) and the noticeable yolk segregation (Fig 5C, 5E and 5G). Wilcox [34] and Fritzenwanker et al. [32] failed to observe the polar bodies and were unable to document *N*. *vectensis’* meiotic chromosome status at fertilization. Finding no polar bodies, Wilcox [34] suggested that the meiosis is completed in the female body, as is true for other cnidarians, ctenophores and sea urchins [45, 58–61] as well as for two anthozoan species [62]. Lee and co-authors [35], however, anectodicaly documented (an outcome that was not illustrated) in *N*. *vectensis* the release of two polar bodies from the animal pole into the jelly coat, suggesting that the polar bodies are displaced after the expansion of the eggs’ jelly. This is also supported by the notion of Hand and Uhlinger [27] that the jelly coat in *N*. *vectensis* is preserved until the release of planulae from it, and Noda and Kanai [63] observation that the polar body in the hydrozoan *Pelmatohydra robusta* is found inside a “mucous-like substance”. In contrast, we documented that in *N*. *vectensis* the polar bodies are shed into the space formed between the external edge of the oolemma and the inner edge of the jelly coat (Fig 6B–6D and 6K).

The most important event occurring during the fertilization process is the cortical reaction, during which the perivitelline space, which separates the egg’s vitelline membrane from the ooplasm, is formed. This is a mechanism that protects the egg from being penetrated by multiple sperm (polyspermy). The basis of the cortical reaction is the hydration of the polysaccharides contained in the cortical granules [64, 65]. In *N*. *vectensis* the cortical layer of the egg is highly loaded with mucopolysaccharides, which start to develop at the onset of vitellogenesis and expand during the growth of the oocyte. By the end of vitellogenesis and during the oocytes’ maturation, the entire surface of the outer cortex of the oocyte becomes rough. After fertilization the cortical layer is preserved, as is typical for many species of sea anemones [42]. In *N*. *vectensis* it hardens and becomes smooth (Fig 6L), except in the area of the animal pole, where the cortical granules are inserted into the space between the outer cortex’s edge and the internal edge of the jelly coat (Fig 6J and 6K). While in *N*. *vectensis* we did not observe the typical cortical reaction accompanied by the formation of the large perivitelline space and the fertilization envelope, we did observed the formation of space between the superficial edge of egg’s surface and the internal edge of the jelly coat at the site of sperm’s penetration. The polar body was ejected exactly into this area (Fig 6B–6D and 6K). Thus, it can be assumed that based on the animal pole’s limited space, this space is similar to the perivitelline space that exists in the fertilized eggs of other animals. Similarly, Clark and Dewel [62] described the cortical reaction in the anthozoan *Bunodosoma cavernanta* as occurring only at the animal pole. Obviously, eggs in *N*. *vectensis* might have a similar mechanism meant to prevent the penetration of more than one sperm into the area where the female pronucleus is located. While several spermatozoa can penetrate in *N*. *vectensis* into an egg simultaneously (physiological polyspermy) (Fig 6l), it is assumed that there is no mechanism to prevent polyspermy in any part of the yolk membrane except for the animal pole, similarly to documentations in other cnidarian species [63, 66, 67] and in siphonophores [68].

Here, we observed 2–4 spermatozoa heads inside *N*. *vectensis* fertilized eggs, while authors of [35] observed numerous spermatozoa on the egg surfaces at the pronucleus site in naïve eggs and single spermatozoa in other areas of the egg surfaces. Such a phenomenon is usually followed by the lysis of the excess sperm heads [64, 69]. As embryogenesis was not studied here no further information is available.

As mentioned earlier, the developing oocytes are surrounded by two types of gastrodermal cells: vacuolated glandular cells and trophocytes; the latter play an important role in the nutrition of oocytes during their growth [5]. The gastrodermal glandular cells accompany germ cells at all stages of oogenesis and probably they form the substances of the jelly coat when the oocytes ovulate into the gastric cavity. This is evidenced by similar tinctorial properties of the glandular cells and the jelly coat (Figs 4B, 6B and 6G). Levitan et al. [9] using in situ hybridization method shown that the mesenterial glandular cells synthesize mucin 5B, which is the mains component of the jelly coat. We have established that the gastrodermal glandular cells produce different polysaccharides, including those resistant to amylase treatment. Similar results were obtained for several species of sea urchin [70, 71] where the extracellular jelly coats consist of neutral and acid mucopolysaccharide-protein complexes. The neutral mucopolysaccharides are amylase-resistant, but the functions of the sulfated polysaccharides of the jelly coat, as their biosynthesis, remain unknown. However, it is believed that they are responsible for inducing the species-specific acrosome reaction necessary for binding the sperm and fusing with the egg [72,73].

Immediately after spawning no oocytes with karyosphere were documented, though numerous previtellogenic and vitellogenic oocytes were seen within the gonadal region. These developing oocytes are probably the source of the next generations of shed eggs, an asynchronous type of germ cell development. Similarly, an asynchronous development of germ cells has been noted in the hydrozoan *Clytia hemisphaerica*, which can spawn daily in the laboratory [74]. Since there are no oocytes with karyosphere in the gonadal region of *N*. *vectensis* immediately after spawning, we can assume that these oocytes have not completed their growth, did not reach a definitive size and could not begin the maturation process. In fish and amphibians only oocytes of definitive sizes acquire competence to begin maturation while responding to environmental and hormonal stimuli. It would be further interesting to study the effects karyospheres impose on the synthesis and accumulation of nutrients in *N*. *vectensis* oocytes throughout the maturation period, and it would be interesting to identify the mechanisms that initiate the maturation process and regulate the sex cells’ competence to respond to the environmental factors. It would also be interesting to determine the potential fecundity of *N*. *vectensis* and the source of its recruited germ cells for use in the extended breeding season under laboratory conditions.
